# Dynamic contact area ratio in shoulder instability: an innovative diagnostic technique measuring interplay of bony lesions

**DOI:** 10.1007/s00167-019-05816-5

**Published:** 2019-12-05

**Authors:** Hanneke Weel, Peter R. Krekel, Nienke Willigenburg, W. Jaap Willems, Pietro Randelli, Riccardo Compagnoni, Derek F. P. van Deurzen

**Affiliations:** 1 Onze Lieve Vrouwe Gasthuis Oost, Amsterdam, The Netherlands; 2 Clinical Graphics, Delft, The Netherlands; 3 Amsterdam University Medical Centres, Amsterdam, The Netherlands; 4 Lairesse Kliniek, Amsterdam, The Netherlands; 5 Laboratory of Applied Biomechanics, Department of Biomedical Sciences for Health, Università degli Studi di Milano, Via Mangiagalli 31, 20133 Milan, Italy; 6 1° Clinica Ortopedica, ASST Centro Specialistico Ortopedico Traumatologico Gaetano Pini-CTO, Piazza Cardinal Ferrari 1, 20122 Milan, Italy

**Keywords:** Shoulder joint, Anterior instability, Shoulder dislocation, Imaging, Diagnostic

## Abstract

**Purpose:**

The hypothesis of this study is that Dynamic Contact Area Ratio of the humerus and glenoid, measured with CT scans, is significantly reduced in patients with anterior shoulder instability compared to the Dynamic Contact Area Ratio in a control group of people without shoulder instability.

**Methods:**

Preoperative CT scans of patients who underwent surgery for anterior shoulder instability were collected. Additionally, the radiologic database was searched for control subjects. Using a validated software tool (Articulis) the CT scans were converted into 3-dimensional models and the amount the joint contact surface during simulated motion was calculated.

**Results:**

CT scans of 18 patients and 21 controls were available. The mean Dynamic Contact Area Ratio of patients was 25.2 ± 6.7 compared to 30.1 ± 5.1 in healthy subjects (*p* = 0.014).

**Conclusion:**

Dynamic Contact Area Ratio was significantly lower in patients with anterior shoulder instability compared to controls, confirming the hypothesis of the study. The findings of this study indicate that calculating the Dynamic Contact Area Ratio based on CT scan images may help surgeons in diagnosing anterior shoulder instability.

**Level of evidence:**

III.

**Electronic supplementary material:**

The online version of this article (10.1007/s00167-019-05816-5) contains supplementary material, which is available to authorized users.

## Introduction

Anterior shoulder instability (ASI) has an incidence of about 2% in the general population [14, 25, 30, 32]. Most dislocations occur in the antero-inferior direction, causing detachment of the anterior labrum, affection of the anterior-inferior glenoid rim and an impact fracture of the postero-superior area of the humeral head, called Hill–Sachs lesion. Shoulder instability has relevant clinical consequences, with limits in return to sport and overhead activities [29, 32]. Recurrent dislocations can also lead to additional injuries on the soft tissues and bony structures, both in the anterior glenoid rim and humeral head [31]. Arthroscopic anterior labrum repair is actually considered the gold standard treatment in patients with labral lesions, but this procedure has shown a high recurrence rate in patients with additional significant bony lesions [7]. In these patients, usually a different type of surgery is performed, like the open Latarjet [11] or other bone block procedure [5, 13].

The influence of bony lesions in recurrences is debated, and many authors correlate instability to the interplay between the bone defect on the glenoid and the humeral head [4, 6–8, 10, 12, 23, 31, 33]. In recent years, many classifications have been proposed to detect and describe these defects such as plain radiography, magnetic resonance imaging (MRI), plain CT, CT with 3-dimensional reconstruction [7, 15, 20, 22, 23, 28], as well as measurement during arthroscopy [6, 7]. Actually, no method is uniformly used in clinical practice to measure the exact extent and orientation of the bony defects of glenoid and humeral head. Above all there is no consensus on how large a glenoid or humeral defect should be to prefer a bony procedure to a less invasive arthroscopic soft tissue repair. Scientific evidence on what amount of bone loss needs which augmentation would be of great value in choosing the optimal surgical intervention.

The results of a dynamic 3-dimensional modality based on plane CT scans were analysed. A ratio was proposed, Dynamic Contact Area Ratio (DCAR) as a novel way to detect the engagement and loss of contact area between glenoid and humerus. The hypothesis of the study is that the preoperative DCAR is significantly lower in patients with anterior shoulder instability compared to the healthy control subjects. This leads to more knowledge of the bony defects, helping clinicians in deciding which (surgical) procedure to perform in patients with anterior shoulder instability.

## Materials and methods

Preoperative CT scans of the patients with documented anterior shoulder instability who were surgically treated in the same hospital (OLVG) in the Netherlands between 2006 and 2012 were collected.

### Patients

The records of all patients with anterior instability (reported by an orthopaedic surgeon), with planned or completed surgical treatment were screened. Patient characteristics (gender, age and side of instability) were collected, as well as the performed surgical procedure. Inclusion criteria were anterior shoulder instability and having a pre-operative CT-scan available. Exclusion criteria were previous shoulder surgery, hereditary exostoses, and CT-scans with arthrography.

### Controls

The radiology database was searched for records with plane CT-scans of shoulders performed for other reasons than anterior shoulder instability. Inclusion criteria were CT-scan of the shoulder, and an age younger than 50 years to exclude changes of the joint surface due to osteoarthritis. Exclusion criteria were shoulder instability, glenoid- or humeral fractures, a history of shoulder surgery, and congenital deformities including glenoid dysplasia or gleno-humeral hereditary exostoses.

### Calculating contact surface/CT measuring

Three-dimensional models of scapulae and humeri from the CT scans of patients and controls using Articulis software, Clinical Graphics, Delft, The Netherlands were extracted [18, 19]. CT scans had a square in-slice resolution between 0.2 and 0.4 mm and a slice thickness between 1 and 3 mm. Glenohumeral function was simulated by rotating and translating the humerus model relative to the scapula in all the collected CT scans, with a previously described and validated kinematic model [18, 19]. The DCAR values are calculated by a mathematical algorithm which is 100% reproducible. When provided with identical inputs, the algorithm will always give identical output values. As a result we cannot report test–retest reliability measurements.

**Fig. 1:**
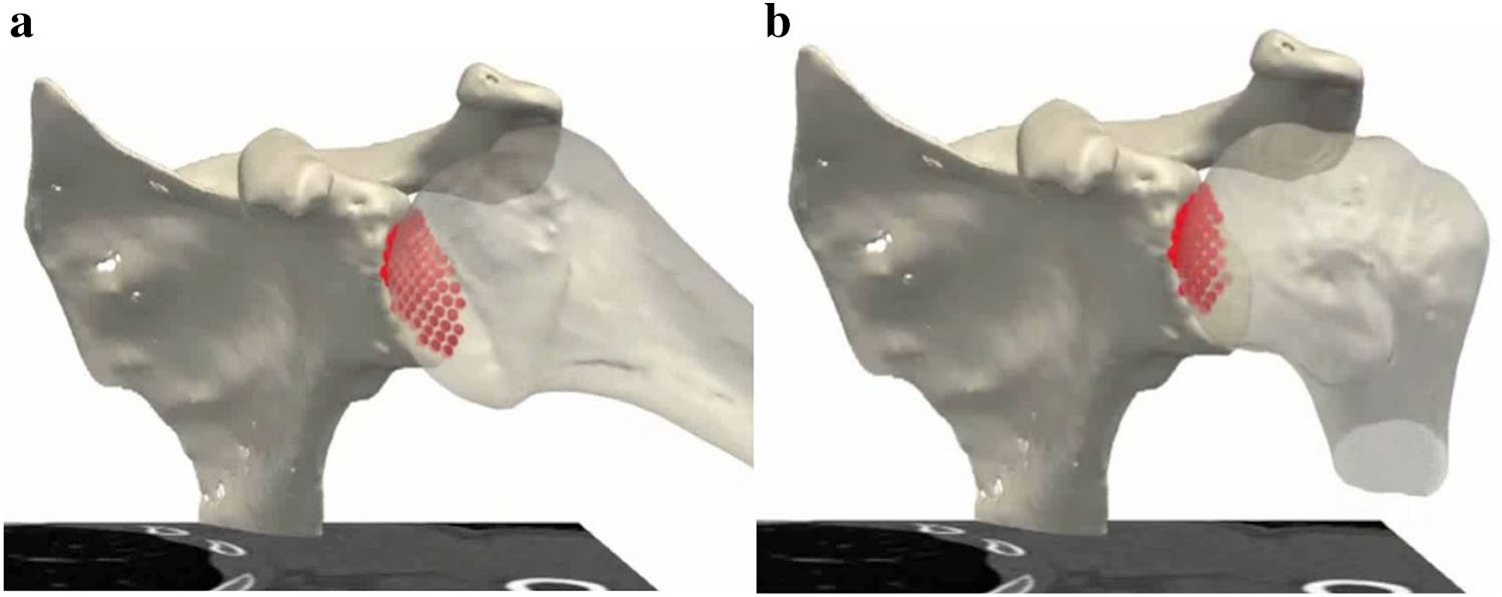
Dynamic CT scan. **a**, **b** Moving humerus with the measured contact area by the software (red dots) during different phases of movement

Flexion, extension, internal and external rotation in 45 and 90 degrees of flexion were simulated (see Fig. 1 and online video). For each step of 1° of each of the motion patterns, the contact area was calculated in mm^2^ between the humeral head and the glenoid. Considering that cartilage is not visible in CT scans the ‘contact area’ was defined as the surface area where the two bone models were within a proximity of 3 mm or less of one another. The Dynamic Contact Area Ratio (DCAR) was defined as the sum of the contact areas of each of the motion steps, after being normalized for humeral head size by dividing the value by the diameter of the humeral head. This diameter was calculated by means of a least-square regression fit of a sphere to the articular surface of the humeral head.

### Ethical approval

As this retrospective study only used data that were already available from procedures performed within standard care, the Dutch Medical Research Involving Human Subjects Act was not applicable. Therefore, the local hospital waived the requirement to obtain informed consent.

### Statistical analysis

Statistics were performed in SPSS 22.0. DCAR data were checked for normality using the Kolmogorov–Smirnov and the Shapiro–Wilk test. The DCAR of patients with diagnosed anterior shoulder instability were compared to healthy controls using an independent samples test (Student’s *t* test or Mann–Whitney *U*, dependent on whether the data were normally distributed or not). A *p* value of ≤ 0.05 was considered as statistically significant.

Given the exploratory nature of this study, the authors were not aware of any previous literature on this specific DCAR as outcome. Therefore, relevant parameters for sample size calculation such as the standard deviation and minimal clinically important difference were lacking. Our aim was to use all available CTs that met the criteria for either patient or control group, with a minimum of 16 CTs in each group. This allows for detecting a difference between groups with the magnitude of 1 standard deviation.

## Results

Eighteen patients met the inclusion criteria in the unstable shoulder group. Fifteen patients (83%) were men. The performed surgeries for the treatment of anterior shoulder instability were either an arthroscopic Bankart repair, reattaching the avulsed anterior capsulo-labral complex to the glenoid neck, or, when a glenoid bone loss of 20% was estimated, the open Latarjet procedure with congruent arch modification [9].

Twenty-one controls were found, whereof 9 (43%) were men. In this group, no surgeries of the shoulder were performed. Additional characteristics are presented in Table 1.

**Table 1:** Patient and control characteristics

	Patients (*n* = 18)	Controls (*n* = 21)
Male	15 (83%)	9 (43%)
Age	35.8 (8.5)^a^	34.3 (9.4)^a^
Left side	50%	52%
Type or primary surgery		
Arthroscopic labrum repair	12 (67%)	x
Open Latarjet	3 (17%)	x
No surgery	3 (17%)^b^	21 (100%)

^a^Data in mean (standard deviation)

^b^Planned to receive an arthroscopic labrum repair but finally refused surgery

**Fig. 2:**
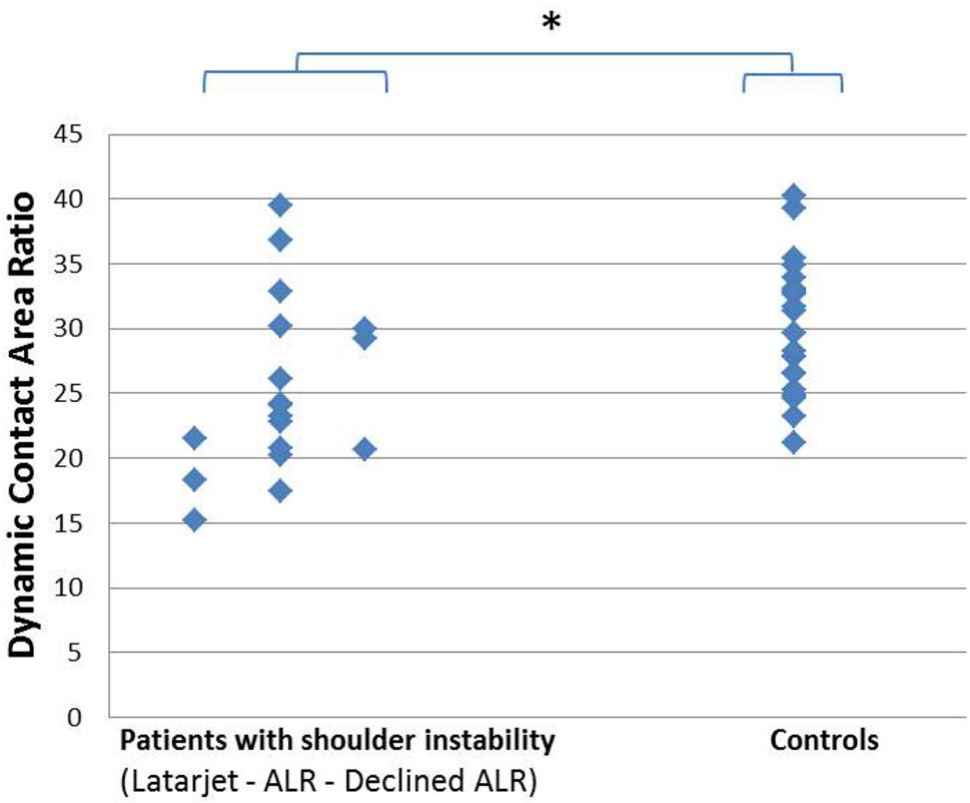
Dynamic Contact Area Ratios of all individuals shown per group. Measured DCAR of each individual (blue diamond shape). ALR: Anterior Labrum Repair. *Significant difference (*p* = 0.014)

DCAR data were normally distributed. The mean cumulative Dynamic Contact Area Ratio (DCAR) measured in the pre-operative CT-scans of all patients with anterior shoulder instability was 25.2 ± 6.7. The control subjects had a significant higher DCAR of 30.1 ± 5.1 (*p* = 0.014). Figure 2 shows the individual DCAR values for each of the participants. The group with shoulder instability is subdivided by the type of surgery they underwent (Latarjet, arthroscopic labrum repair or none). The cohort was too small to perform statistical subgroup analyses.

## Discussion

The most important finding of the present study was that the DCAR based on pre-operative CT scans was significantly lower in patients who needed surgical treatment for anterior shoulder instability compared to the control subjects without shoulder instability, confirming the hypothesis. This pilot-study evaluated the applicability of a novel tool that uses a 3-dimensional simulation model to calculate the dynamic contact area between the glenoid and the humerus.

While the patient cohort was too small to perform statistical subgroup analyses, DCAR values seemed lower in patients treated with an open Latarjet procedure, than in patients who underwent (or were scheduled for) an arthroscopic Bankart repair. This would be plausible, as the Latarjet procedure was only performed in patients who were deemed to have a “significant large” glenoid defect. However, the observed range of DCAR values within (sub)groups reflects the complexity of setting cut off values for use in individual patients, which warrants further research.

A limitation of this study is the retrospective design in which patient characteristics such as the level and type of sports participation, that have shown to be prognostic factors for surgical success [26, 27], were not collected. Also the outcome after surgery could not be completely investigated, due to too much missing data.

A further consideration is that in the radiological workup for shoulder instability, imaging soft tissue is still recommended to be performed by MRI arthrography [1], although in clinical practice, CT-scans are advised to analyse bony lesions [24, 31]. In the original planning of surgery for the reviewed group of patients the choice for either a soft tissue Bankart repair or Latarjet was based on non-quantitative judgement of the bone defect in preoperative MRI-and CT-scans.

This study showed a significant difference in bony contact area between stable and unstable shoulders. The difference in DCAR between the healthy and unstable shoulders supports previous findings by Sugaya et al. [31], who found bony loss of the glenoid to be present in most shoulders after dislocation. Several techniques were designed to measure bone loss, for example the above named Sugaya method [31]. Their technique is based on quantifying the size of the loose glenoid fragment and comparing it to the glenoid fossa (being > 20%, 5–20% and < 5%, respectively). Based on a cadaveric study, Itoi et al. [16] recommend making a West Point View followed by a CT scan if this is equivocal or hard to obtain due to pain or apprehension. The PICO method [3] draws a best-fit circle on the inferior portion of the uninjured, contralateral glenoid, which subsequently is superimposed onto the injured side. The area missing in the circle (the bony defect) is then divided by the area of the best-fit circle to estimate the percentage glenoid bone loss. An MRI-based method using OsiriX has also been suggested for measuring bony defects of the glenoid [22].

The lack of consensus on cut-off values for bone loss may be due to the inherent difficulty when trying to calculate the dimensions of bone that is missing, rather than measuring structures that are remaining. The currently presented method not only focuses on the intact structures but also determines the positions in which the humeral head is still in contact with the glenoid during simulated three-dimensional directions. In this way, the possible engagement of Hill Sachs lesions with the anterior glenoid rim in abduction-external rotation can be assessed as well. This method of calculating a DCAR is, therefore, more comprehensive than the currently available methods that only measure the loss of bone [2, 3, 16, 17, 21, 22, 31]. It also differs from the glenoid track method [10, 33] by not only calculating the remaining bone contact of glenoid and humerus, but also defining the remaining contact area during simulated range of motion. The on–off track method [10] additionally measures the interplay of the glenoid and humerus, with an on- or off track humerus lesion. Although this concept is promising, the technique is complicated for clinical usage. With the DCAR method, the bone contact area is easily measured during a wide range of motion and thus auspicious mimicking the reality of an unstable shoulder.

DCAR uses a plain CT-scan, which is a fast and widely available diagnostic tool. As soft tissue plays an important role in shoulder instability too, there are benefits to the use of MRI rather than CT-scans. Therefore, also making MRI available as input for the DCAR calculation is currently work in progress.

The DCAR method should be validated and compared with other methods, like the “on–off track” method [10]. A prospective study could make it possible to recommend a cut off DCAR-value from where to advise a bony repair instead of a soft tissue procedure.

For clinical practice, integrating the DCAR in a generally available diagnostic system would enable surgeons to choose the proper procedure (no surgery needed, performing a labrum repair or a bony repair) depending on the amount of remaining contact surface area in the injured shoulder joint.

## Conclusion

The DCAR based on standard CT-scans is a potentially promising objective tool that could guide diagnostics in patients with anterior shoulder instability. This method analyses the engagement of humerus and glenoid during shoulder motion, which seems to be valuable for examining the impact of bone loss in shoulder instability.

## Electronic supplementary material

Below is the link to the electronic supplementary material.

Supplementary material 1 (MOV 5947 kb)

## Abbreviations

DCAR: Dynamic Contact Area Ratio
ASI: Anterior shoulder instability
